# Dynamic Tissue—Specific Transcriptome Changes in Response to *Verticillium dahliae* in Wild Mint Species *Mentha longifolia*

**DOI:** 10.3390/plants11050674

**Published:** 2022-03-01

**Authors:** Kelly J. Vining, Iovanna Pandelova

**Affiliations:** Department of Horticulture, Oregon State University, Corvallis, OR 97331, USA; iovanna.pandelova@oregonstate.edu

**Keywords:** mentha, mint, verticillium, disease resistance, wilt, monoterpene

## Abstract

*Mentha longifolia* is a wild mint species being used as a model to study the genetics of resistance to the fungal wilt pathogen *Verticillium dahliae*. We used high-throughput Illumina sequencing to study gene expression in response to *V. dahliae* inoculation in two *M. longifolia* USDA accessions with contrasting phenotypes: wilt-resistant CMEN 585 and wilt-susceptible CMEN 584. Roots and stems were sampled at two early post-inoculation time points, four hours and twenty-four hours, and again at ten days and twenty days post-inoculation. Overall, many more genes were differentially-regulated in wilt-resistant CMEN 585 than in wilt-susceptible CMEN 584. The greatest numbers of differentially expressed genes were found in the roots of CMEN 585 at the early time points. Specific genes exhibiting early, strong upregulation in roots of CMEN 585 but not in CMEN 584 included homologs of known plant defense response genes as well as genes involved in monoterpene biosynthesis. These genes were also upregulated in stems at the later time points. This study provides a comprehensive view of transcription reprogramming in Verticillium wilt-resistant mint, which will be the basis for further study and for molecular marker development.

## 1. Introduction

**Figure 1:**
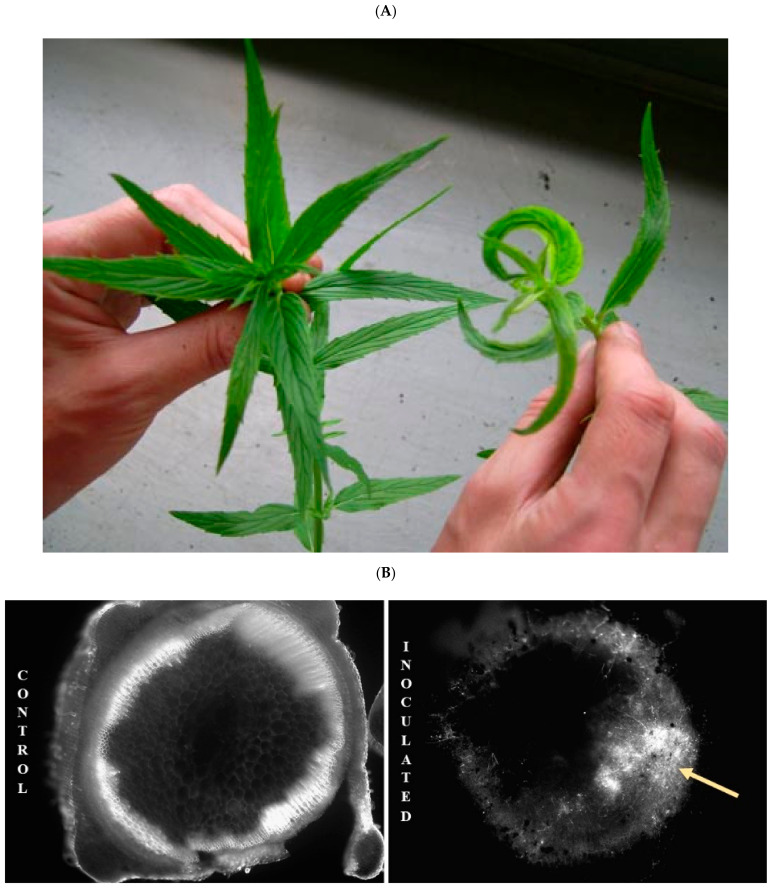
Verticillium wilt disease symptoms in mint. (**A**). Left: uninoculated *M. longifolia*. Right: *V. dahliae*-inoculated *M. longifolia* displaying chlorosis and crescent leaf symptoms. (**B**). *M. longifolia* stem cross-sections were taken 20 days after plants were root dipped in either water (control) or a spore suspension of GFP-labeled *V. dahliae* (inoculated). Stem sections were incubated on water/agar for several days before examining by fluorescent microscopy and imaging. The arrow shows GFP-labeled fungal hyphae.

Vascular wilt disease caused by the soil-borne deuteromycete fungus *Verticillium dahliae* is a devastating disease of commercial peppermint (*Mentha × piperita*). The fungus invades through the root system and proliferates in the xylem. Symptoms of Vertcillium wilt disease in mint range from mild chlorosis and “crescent leaf” (Figure 1) to wilting and death of plants. *V. dahliae* is a widespread plant pathogen, affecting annuals, perennials, and woody plants. As such, Verticillium wilt disease has been studied in many different crops, including cotton, lettuce, and the Solanaceae [1,2,3,4,5,6,7,8].

Genetic resistance to Verticillium wilt was first mapped in tomato, and two linked genes at the mapped locus, *Ve1* and *Ve2*, were cloned in 2001 [9,10]. *Ve1* but not *Ve2* was shown to confer resistance to tomato Verticillium wilt. Subsequent studies identified positive regulators of *Ve1* as well as downstream signaling components in the *Ve1*-mediated pathway.

Mint is an excellent model system for the study of Verticillium wilt disease. Mint plants produce abundant stems with relatively uniform internode lengths, making experimental replication straightforward. Plants have square stems, with vascular bundles located at each corner. When fungal hyphae clog xylem of susceptible mints, expansion of apical leaves is inhibited, causing the classic “crescent leaf” symptom (Figure 1). The crescent leaf curves toward the infected stem corner, meaning that specific infected vascular bundles are readily observed.

*M. longifolia* has been developed as a diploid model system to study mint genetics, including Verticillium wilt resistance, as the available germplasm for this species represents a broad range from resistance to susceptibility [11]. While much has been learned about the genetics of response to *V. dahliae* infection in other crops, it has become clear that there is not one uniform set of genes to interrogate in all plant genera. Therefore, the aim of this study is to identify the most important genetic components of Verticillium wilt resistance in mint.

To this end, we present a comprehensive profile of gene expression in roots and stems of two South African *M. longifolia* accessions representing extremes of Verticillium wilt resistance vs. susceptibility. Wilt-resistant accession CMEN585 belongs to *M. longifolia* L. subsp. *capensis,* and its essential oil is predominantly pulegone. Wilt-susceptible accession CMEN584 is *M. longifolia* L. subsp. *polyadena* and produces mainly carvone [11]. By sampling root and stem tissues at four post-inoculation time points, we captured transcripts from early in the infection process to the beginning of symptoms display.

## 2. Results

### 2.1. Transcriptome Data Summary

Transcriptome sequencing was done on RNA samples isolated from roots and stems of either water- (control) or *V. dahliae* spore-inoculated CMEN 585 and CMEN 584 plants. Samples were collected at early (4 and 24 h post-inoculation (hpi)) and late (10 and 20 days post-inoculation (dpi)) stages of *V. dahliae* infection.

Sequence data obtained per replicate sample ranged from 2.6–13.4 Gb, the equivalent of 50.2x–256.2x coverage of gene annotations in the reference genome (Supplemental Table S1). Transcriptome coverage depths per biological replicate were similar between CMEN 585 and CMEN 584. The 10-dpi and 20-dpi time points had ~15–20x lower coverage depth per replicate than the 4-hpi and 24-hpi time points.

### 2.2. Overview of Differentially-Expressed Genes

**Figure 2:**
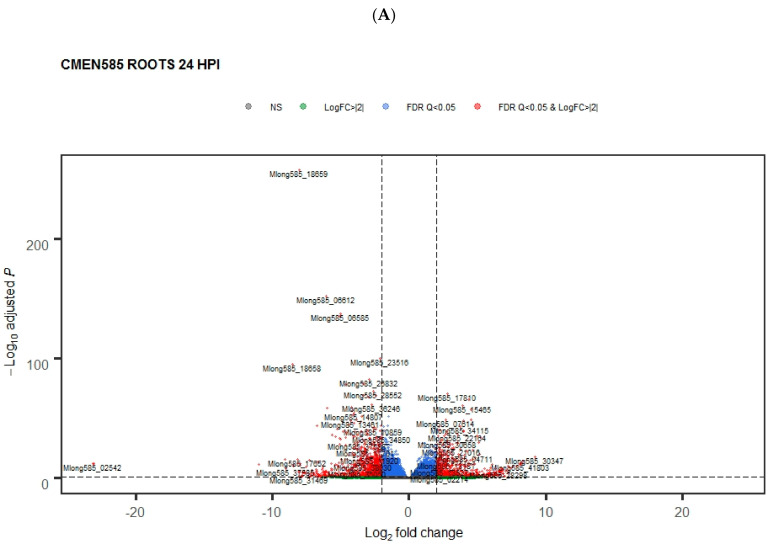

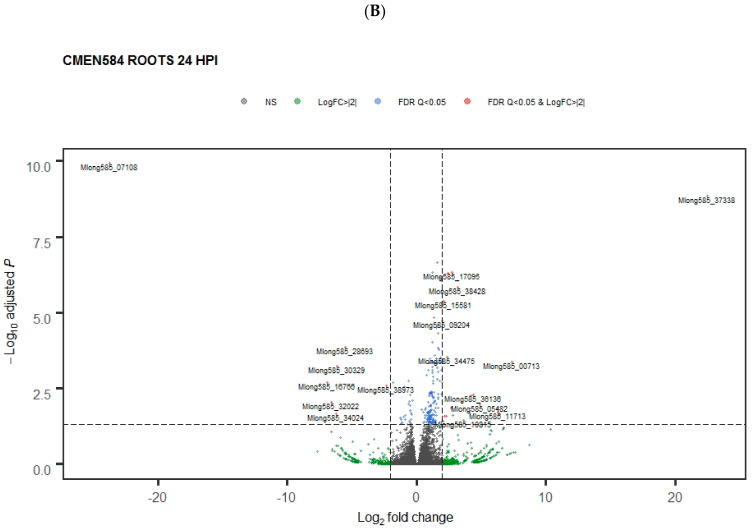
More genes are differentially regulated in roots of CMEN 585 than in roots of CMEN 584 early after exposure to *V. dahliae*. The volcano plots show genes that are significantly upregulated or downregulated (red data points) in (**A**). Resistant *M. longifolia* CMEN 585 and (**B**) susceptible CMEN 584 at 24 h post-inoculation with *V. dahliae*.

**Figure 3:**
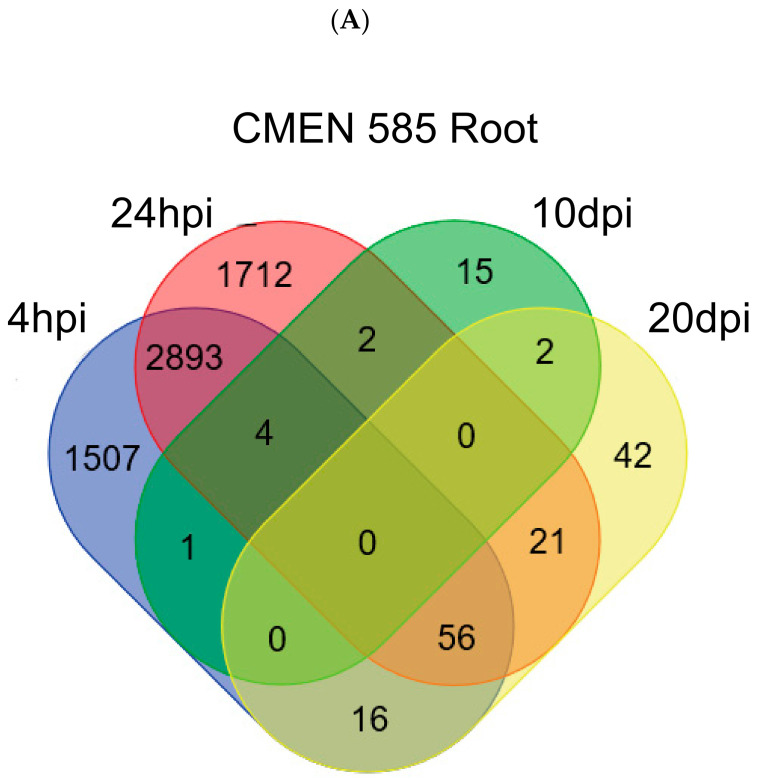

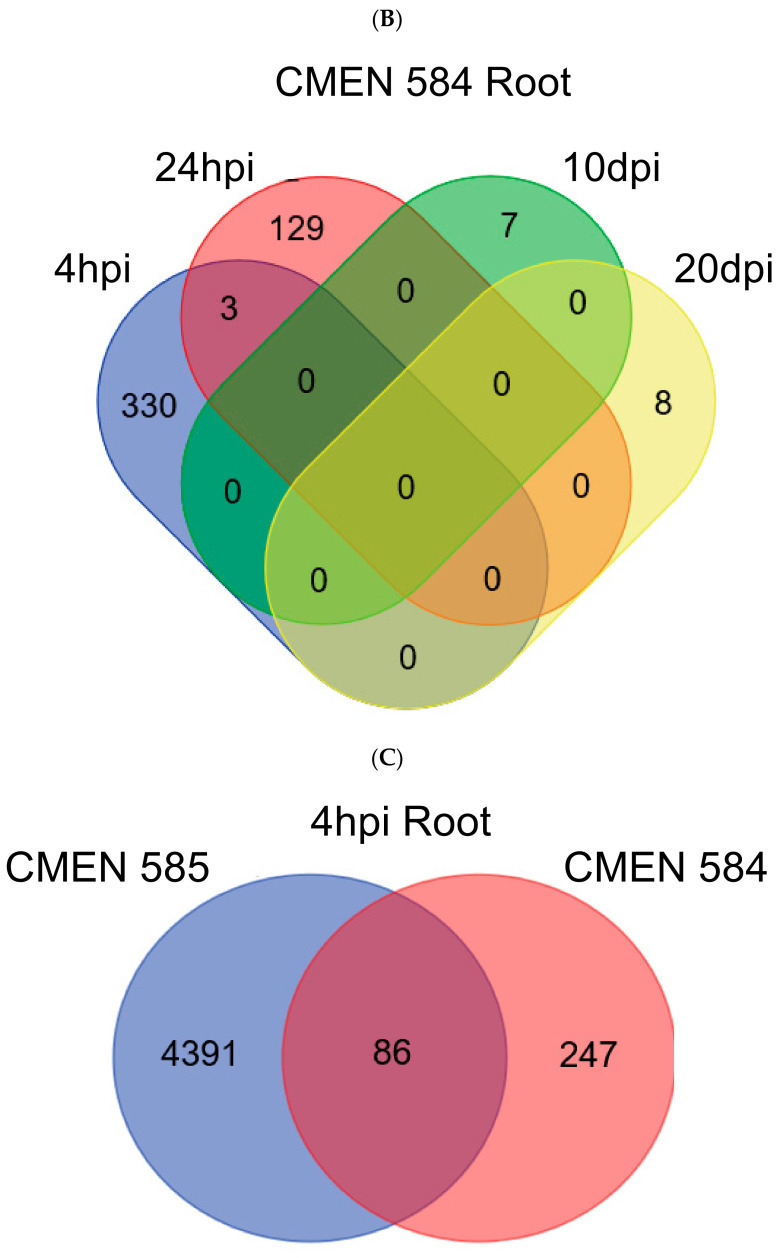
Overlaps among differentially-expressed gene sets in (**A**) CMEN 585 roots across all sampling times (hours post-inoculation (hpi), days post-inoculation (dpi)); (**B**) CMEN 584 roots across all sampling times; (**C**) CMEN 585 and CMEN 584 roots at four hours post-inoculation (hpi).

Pairwise comparisons of gene expression levels between control and inoculated CMEN 585 and CMEN 584 plants were made for each tissue at each time point. The numbers of differentially expressed genes were highly variable between the two mint genotypes as well as across time points, ranging from <10 to >4000 (Table 1, Figure 2 and Figure 3).

No genes in roots or stems of either genotype were consistently upregulated or downregulated across all sampling times (Figure 3). However, the following four genes were upregulated in CMEN 585 roots at three time points from 4 hpi through 10 dpi: Mlong585_01266 (similar to SBT1.9: Subtilisin-like protease SBT1.9 (*Arabidopsis thaliana*)), Mlong585_00612 (similar to LAC14: Laccase-14 (*Arabidopsis thaliana*), Mlong585_16146 (similar to BZIP53: bZIP transcription factor 53 (*Arabidopsis thaliana*)), and Mlong585_16791 (similar to Glucan endo-1,3-beta-glucosidase, acidic isoform PR-Q).

### 2.3. Extensive Transcriptome Reprogramming in Roots of CMEN 585, but Not CMEN 584, at Early Time Points

By far, the greatest numbers of differentially expressed genes were detected in roots of wilt-resistant genotype CMEN 585 at the four-hour (4477 genes) and 24-h (4688 genes) post-inoculation time points (Figure 2, Table 1). Numbers of upregulated and downregulated genes were similar. The most notable overrepresented gene ontology (GO) categories in these gene sets concerned response to biotic stimulus/defense response, transmembrane transport, cell wall biosynthesis, and terpene biosynthesis (Supplemental Figure S1).

In contrast to roots of wilt-resistant CMEN 585, wilt-susceptible CMEN 584 roots showed only a few hundred differentially expressed genes at 4 hpi and 24 hpi (Figure 2, Table 1). As a result, there were few overrepresented GO categories, most of which were related to transmembrane transport. One notable exception was genes regulating biosynthesis of quinones, which were upregulated at 4 hpi in CMEN 584 but not CMEN 585.

### 2.4. Gene Expression in Stems at Early Time Points Differs from Roots

Gene expression patterns in stems at 4 hpi and 24 hpi were markedly different from those of roots (Table 1). CMEN 585 had only 101 differentially expressed genes over both time points, none of which was represented at both time points. At the 4-hpi time point, CMEN 584 had nearly twenty times more differentially expressed genes than CMEN 585, 70% of which were downregulated. GO categories enriched in the downregulated gene set were associated with oxidative stress (response to oxidative stress, GO:0006969; response to toxic substance, GO:0009636) and with biosynthesis of quinones (quinone biosynthetic process, GO:1901663).

### 2.5. Role of Disease Resistance Gene Homologs in Early Response to V. dahliae Infection

**Figure 4:**
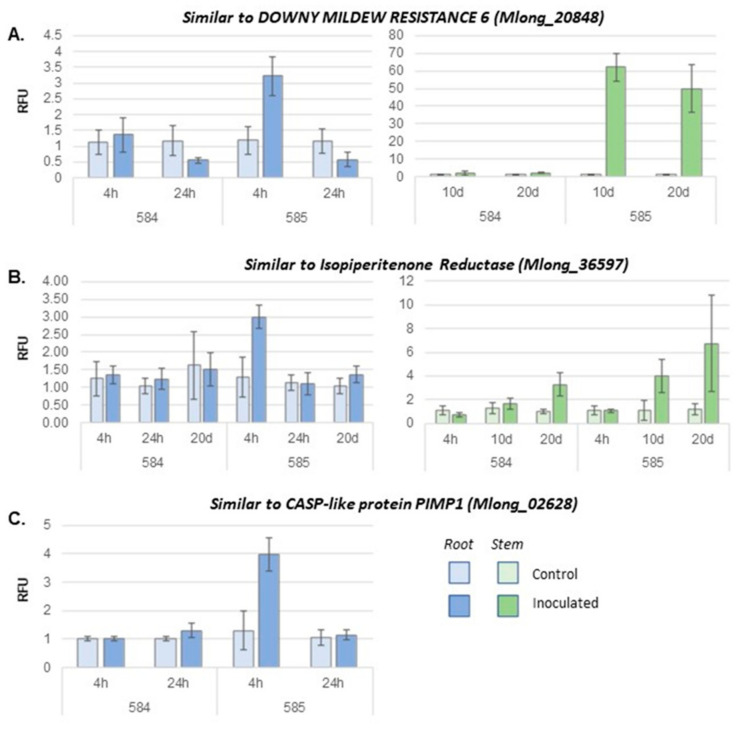
Quantitative RT-PCR (qRT-PCR) results from genes showing upregulation in response to *V. dahliae* inoculation in CMEN 585 but not in CMEN 584. RFU, relative fluorescence units.

Several *M. longifolia* genes with homology to known or implicated plant disease resistance genes showed a pattern of steep upregulation in roots of CMEN 585 and not in CMEN 584 at 4 hpi. This initial spike was followed by a decrease in expression such that at 24 hpi, expression levels in roots in inoculated plants were at or even below levels in controls (Figure 4A,C). Genes showing this pattern included two homologs of *DOWNY MILDEW RESISTANCE 6* (*DMR6*) from *Arabidopsis thaliana*, Mlong585_20848 on chromosome 6 and Mlong585_36694 on chromosome 11; two homologs of CASP-like protein PIMP1, Mlong585_02628 on chromosome 1 and Mlong585_14355 on chromosome 4; and two chitinase-like genes, Mlong585_18213 and Mlong585_18310 on chromosome 5. These were upregulated relative to controls 3.6–5.9-fold in CMEN 585 roots and 5.4–8.4-fold in stems. In CMEN 584, these genes were not found to be differentially expressed in either tissue. Mlong585_18213 is adjacent to three other chitinase homologs that were not differentially expressed.

Besides the two chitinase-like genes, three of the most highly upregulated genes in CMEN 585 at 4 hpi were located near one or more NBS-LRR genes on chromosome 5. One of them, Mlong585_17212, is a homolog of the *Prunus armeniaca* major allergen *Pru ar 1*. This gene was adjacent to five other *Pru ar 1* homologs and nearby homologs of two other major plant allergen genes: *MALD1* (*Malus domestica*) and *PRUA1* (*Prunus avium*).

### 2.6. Role of Monoterpene Biosynthesis-like Genes in Response to V. dahliae

A cluster of 13 monoterpene biosynthesis gene homologs annotated as “isopiperitenone reductase (ISPR)-like”, located in a region on chromosome 11 between 2.1 Mb and 3.3 Mb, showed contrasting early expression profiles in CMEN 585 and CMEN 584. Two of these genes, Mlong585_36604 and Mlong585_36605, had the highest homology to the canonical peppermint ISPR gene that is expressed in mint glandular trichomes (Vining et al., in press). The ISPR-like genes showed a range of relative expression levels from low to very high, following a pattern like that of the plant disease-resistance genes (Figure 4B). They were upregulated 1.56–3.86-fold in inoculated roots relative to controls at 4 hpi, after which expression decreased and was similar to controls at 24 hpi. At 10 dpi, in both CMEN 585 and CMEN 584, expression of the genes was not significantly different from controls. At 20 dpi, three of the ISPR genes, namely Mlong585_36604, Mlong585_36587, and Mlong585_36597, were significantly upregulated relative to controls (Figure 4B).

In stems at the early time points, none of the ISPR or ISPR-like genes was significantly differently regulated relative to controls in either genotype. At 10 dpi, eleven of the genes were significantly upregulated in stems of inoculated plants relative to controls in CMEN 585, but none were upregulated in CMEN 584. By 20 dpi, ten of the genes were upregulated in both CMEN 585 and CMEN 584.

**Figure 5:**
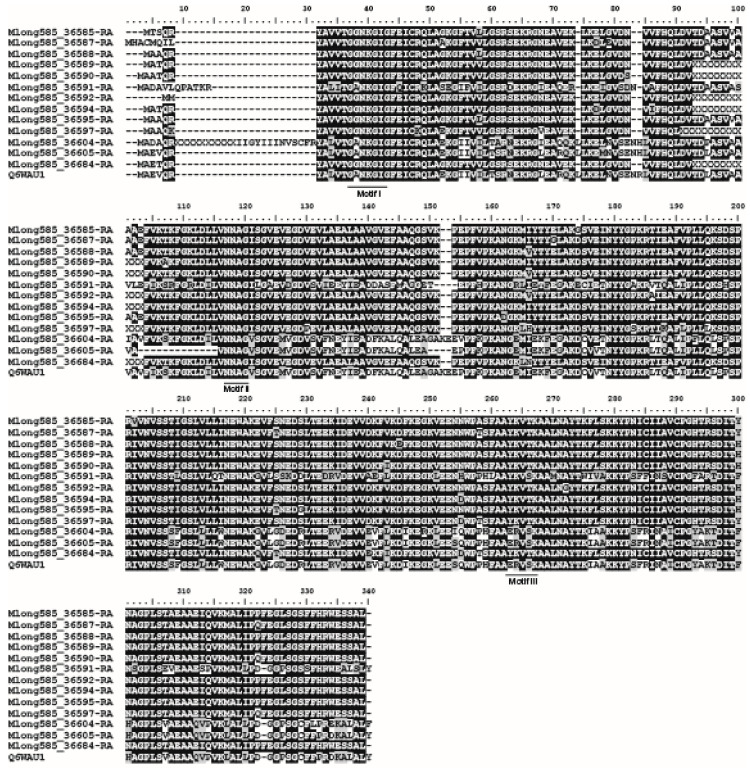
Alignment of 13 *M. longifolia* isopiperitenone reductase amino acid sequences with the canonical sequence (QSWAU1) from peppermint [12]. X characters indicate ambiguous nucleotides in the *M. longifolia* reference genome. Conserved coenzyme motif (Motif I), NNAG site (Motif II), and active site motif (Motif 3) are underlined.

Alignments of the ISPR genes and their predicted proteins showed that they differed from each other by SNPs and indels that translated into amino acid sequence differences, including at the identified coenzyme binding and active site motifs (Figure 5) [12]. However, ambiguous nucleotides (Ns) in these genes in the *M. longifolia* reference genome precluded identification of all possible polymorphisms.

### 2.7. Transcriptional Reprogramming Extends to CMEN 585 Stems at Later Sampling Points

Gene expression was profiled in roots and stems at 10 dpi and 20 dpi to compare gene expression profiles early in infection with those later in infection. Consistent with the earlier time points, CMEN 585 had more differentially expressed genes than CMEN 584 at the 10-dpi and 20-dpi sampling points. However, in contrast to the early sampling points, the later time points showed more differentially expressed genes in stems than in roots of both genotypes.

**Figure 6:**
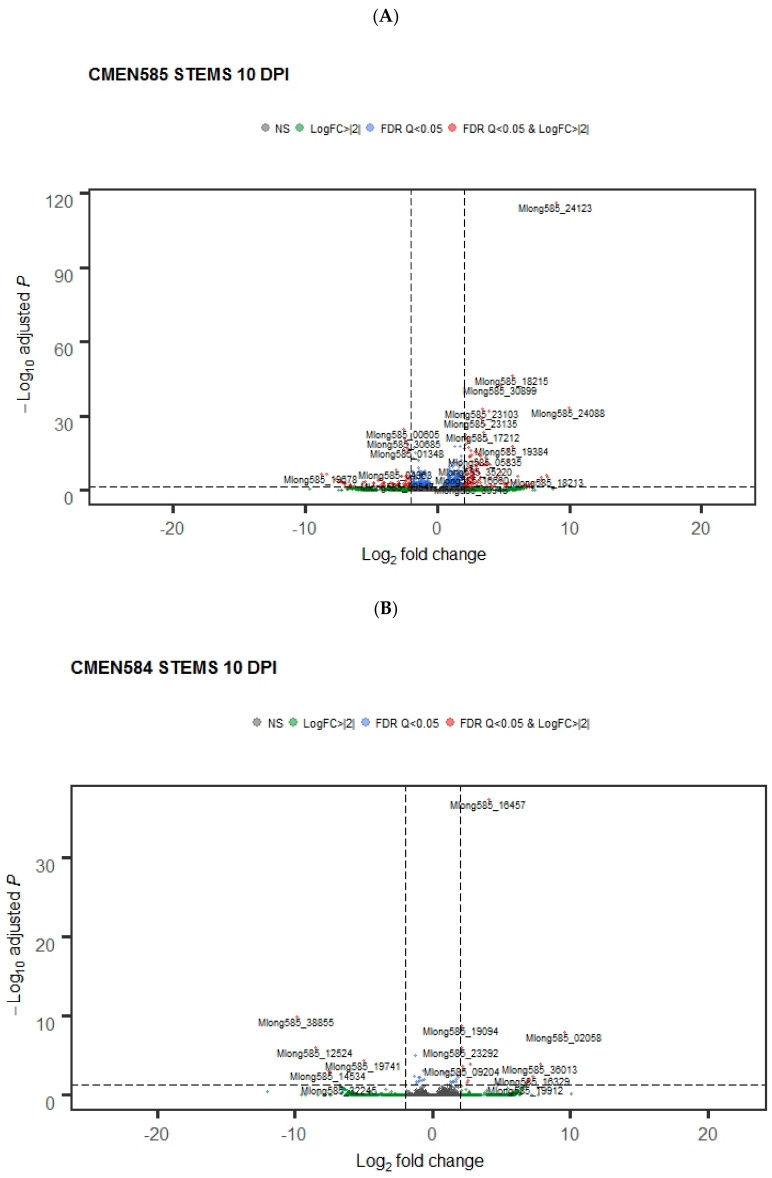
Volcano plots showing differentially expressed gene profiles in stems of (**A**) CMEN585 and (**B**) CMEN584 at 10 and 20 days post-inoculation.

In roots, the number of differentially expressed genes was drastically lower at 10 dpi and 20 dpi relative to 4 hpi and 24 hpi (Figure 6). For CMEN 584, the numbers of differentially expressed genes at the later time points were fewer than ten. CMEN 585 roots had 24 differentially expressed genes at 10 dpi and 137 differentially expressed genes that were predominantly up-regulated at 20 dpi. Overrepresented GO categories in the latter group were associated with transmembrane transporter activity, manganese ion binding, and nutrient reservoir activity.

In stems at 20 dpi, CMEN 584 had more up-regulated than down-regulated genes. Overrepresented GO categories were associated with secondary metabolic processes and cellular amino-acid-derivative metabolic processes. Transmembrane transport was also overrepresented and included both up- and down-regulated genes.

As in roots at the earlier time points, CMEN 585 had noticeable transcriptional reprogramming in stems at the later time points. At 24 hpi, there were fewer than 70 differentially expressed genes in stems, the majority of which were down-regulated. At 10 dpi, there were 561 differentially expressed genes, more showing up-regulation. The upregulated gene set was enriched for GO categories associated with terpene and monoterpene synthesis. The defense response GO category was also overrepresented, including pathogenicity-related genes, such as chitinases, glucanases, and thaumatin. In 20-dpi stems, overrepresented GO categories in the differentially expressed gene set included oxidoreductase activities, monooxygenase activity, and heme binding.

Some of the most highly upregulated genes at the 10-dpi time point in CMEN 585 were upregulated in both roots and stems. One example is a set of three thaumatin-like genes on chromosome 8 that were upregulated ~2–9-fold. These genes are located at a gene cluster in a 3-Mb region on chromosome 8 that also includes NBS-LRR genes, a MYB transcription factor, and two genes predicted to be involved in increased DNA methylation (IDM1). In CMEN 584, these genes were downregulated in roots 0.5–2.4-fold relative to controls and in stems were upregulated only 0.33–1-fold.

## 3. Discussion

*M. longifolia* accessions CMEN 585 and CMEN 584 are part of a long-term mint research program aimed at building foundational mint genetic and genomic resources. While both accessions were collected from South Africa and superficially appear very similar, they are classified as different subspecies, and they show many contrasting phenotypes, with Verticillium wilt resistance being one that was identified early in the research program [11]. The two plants have been frequently used in subsequent screening trials as resistant and susceptible checks. Dissection of inoculated plants has shown that the fungal hyphae enter stems of susceptible CMEN 584 between 10–20 dpi, coincident with the first noticeable wilt disease symptoms, such as typically mild crescent leaf and/or chlorosis (unpublished data). Hyphae are also sometimes observed in stems of CMEN 585, but symptoms are typically mild or absent. These observations led to the first experiment in the present study, aiming to answer questions about which genes were differentially expressed in the two plant genotypes during the 10-dpi–20-dpi time window and potentially played a role in limiting disease symptoms in CMEN 585.

Analysis of the data from the first experiment led to questions about gene regulation early in the infection process that might influence later gene expression. In the interim, a new Illumina sequencing instrument with higher data output became available. The early gene expression experiment included both more biological replicates and up to ~20x higher coverage depth per biological replicate of mapped reads over the annotated transcriptome. Some of this difference in coverage depth can clearly be attributed to sequencing done on different Illumina instruments, but fungal proliferation within plant tissues, especially in roots, may also be a factor. *V. dahliae* is able to colonize roots of both CMEN 584 and CMEN 585 although the latter typically shows no or mild disease symptoms.

Lower transcriptome coverage depth may have contributed to the relatively lower numbers of differentially expressed genes identified at the later time points but does not entirely explain the overall wide variability in numbers of differentially expressed genes. Another consideration is the reference genome used in this study, which was generated from CMEN 585. CMEN 585 and CMEN 584 are categorized as different subspecies; CMEN 585 is *M. longifolia* ssp. capensis, while CMEN 584 is *M. longifolia* ssp. *polyadena*. The two accessions have phenotypic differences beyond Verticillium wilt susceptibility, including chemotype and male fertility. Allelic divergence and structural variations may have contributed to lower overall percentage read mapping of CMEN 584 to the CMEN 585 reference, but this also does not explain the variability in numbers of differentially-expressed genes between the genotypes. In many cases, transcriptome coverage depths were comparable between the two genotypes, but numbers of identified differentially-expressed genes were vastly different. For example, in stems at the 24-hpi time point, the median per-replicate coverage depths for CMEN 585 and CMEN 584 were 72.3x and 67.2x, respectively, but only 33 differentially expressed genes were found in CMEN 585, while 630 were found in CMEN 584. Ultimately, while data quantity and within-species genetic diversity may partially explain some of the variation in numbers of differentially-expressed genes, these results also support a biologically based hypothesis: an early, strong response in CMEN 585 roots is crucial to Verticillium wilt resistance in mint, and it is lacking in CMEN 584. CMEN 584 appears to have activation of some crucial resistance responses, such as generation of reactive oxygen species. However, by 20 dpi, CMEN 584 begins to be overwhelmed by the fungus.

Most differentially expressed genes were detected as such at only one or two time points. However, four genes were an exception to this trend, showing up-regulation in CMEN 585 roots from 4 hpi through 10 dpi. These genes had similarity to a subtilisin protease, a laccase, a bZIP transcription factor, and a glucan endo-1,3-beta-glucosidase. Plant subtilases are abundant serine proteases, some of which become glycosylated and accumulate in the extracellular matrix in response to pathogen attack [13,14]. Beta 1,3-glucanases are part of the pathogen-related (PR) protein category, which, along with chitinases, are induced by fungal pathogen attack and degrade fungal cell walls [5]. Both types of genes have been subjects of transgenic approaches to confer disease resistance in a variety of crop species [15,16,17]. This study provides evidence for a role of these genes in a successful *V. dahliae* resistance response in mint.

Since *V. dahliae* enters plants via their roots, we expected to find induction of defense-related genes soon after pathogen inoculation. This was where a crucial difference in gene expression between the two mints was revealed. Verticillium wilt-resistant CMEN 585 had increased expression of sets of genes related to plant defense responses, intercellular signaling, and monoterpene biosynthesis. In contrast, the only functional category of genes upregulated early after inoculation in CMEN 584 roots but not in CMEN 585 roots was that of quinone biosynthesis. CMEN 584 did not simply lack the transcriptome reprogramming that occurred in CMEN585; rather, CMEN584 had an overall different response. Broadly, plant defense consists of a collection of different responses. These can include a hypersensitive response; production of physical barriers, such as papillae or tyloses; and production of antimicrobial toxins [18]. The most effective plant defense responses are usually induced by early molecular recognition of specific pathogens. In the absence of pathogen-specific recognition, plant defense responses can be induced in a more generic way, such as in response to abiotic stress or wounding. Biosynthesis of quinone and derivatives can be considered a generic response, implicated in reactive oxygen species production in response to fungal attack but also in response to herbivory and abiotic stress [19,20,21]. Therefore, the results of this study indicate that CMEN 585 recognizes *V. dahliae* and mounts a rapid, robust defense, whereas CMEN 584 does not. In the tomato-*V. dahliae* pathosystem, the Ve protein plays the recognition role. Mint has *Ve* homologs [22], but the *M. longifolia Ve*1 and *Ve*2 genes were not found to be differentially expressed in either mint genotype at any of the sampling times in this study. Therefore, follow-up work will closely investigate alleles of defense genes that are most likely to be involved in early recognition of *V. dahliae*.

Defense genes showing the greatest early upregulation in CMEN585 roots were homologs of CASP-like protein *Pathogen-Induced Molecular Protein 1* (*PIMP1*) and *Downy Mildew Resistance 6* (*DMR6*). *PIMP1* encodes an integral membrane protein and has been shown in pepper to play a role in both resistance to bacterial pathogen *Xanthomonas campestris* and susceptibility to *Pseudomonas syringae* [23,24]. The *DMR6* gene was first identified and cloned from an Arabidopsis EMS mutant, where *dmr6* mutants lost susceptibility to *Hyaloperonospora arabidopsidis* [25]. Subsequent studies identified partially redundant *DMR6*-like oxygenase genes in Arabidopsis and tomato [26,27] and showed that *dmr6* mutations conferred resistance to a broad range of bacterial, oomycete, and fungal plant pathogens. *DMR6* encodes a 2-oxoglutarate (2OG)-Fe(II) oxygenase that is a negative regulator of salicylic acid (SA) production. SA itself serves as a mediator of intersecting defense signaling pathways, where multiple genes, including *Enhanced Disease Susceptibility* 1 (*EDS*) and *ALD*1, regulate SA synthesis and accumulation, and others, such as *PR1* and *NPR1,* act as signal transducers [28,29,30].

At the later 10-dpi and 20-dpi time points, the most up-regulated genes in CMEN585 were up-regulated in both roots and stems. These included and thaumatin-like genes and genes encoding chitinases. The role of chitinases in plant resistance to fungal pathogens has been known since the 1980s [31]. A number of recent publications have reported genome-wide surveys of the chitinase gene family in plants as diverse as *Eucalyptus grandis* [32], *Brassica* spp. [33,34], *Camellia* (tea) [35], and the Asian evergreen shrub *Ammopiptanthus nanus* [36]. This study adds to the body of knowledge of plant chitinase diversity and expression as a defense against fungal pathogens.

Plant defense genes in CMEN 585 showed an early, strong pulse in expression in roots followed by a decrease within 24 h post-inoculation. In some cases, this was followed by an expression increase in stems during the 10–20-day post-inoculation time period. This indicates a long-range signaling cascade spanning roots and stems, likely involving SA, that ultimately results in systemic acquired resistance (SAR).

Terpenes are the largest, most diverse class of plant secondary metabolites, and the fact that terpene biosynthesis itself is a plant defense response is well known [37]. Work with peppermint (*M.* × *piperita*) has shown that inoculation of roots with *Rhizobacteria* can increase both salicylate and jasmonate production while also increasing phenolic content and monoterpene essential oil production [38,39]. Some breeding programs are already targeting increased terpene production as a disease-resistance breeding strategy [40]. This study implicates a particular monoterpene biosynthesis gene in mint resistance to *V. dahliae*: isopiperitenone reductase. A cluster of ISPR-like genes spanning ~1 Mb on chromosome 11 showed upregulation in CMEN585 roots at 4 hpi and in stems at 20 dpi. Sequence divergence among these paralogous genes make it likely that RNA-seq reads aligned to their true loci of origin rather than nonspecifically. In fact, we were able to target individual ISPR-like genes in the cluster with qRT-PCR, indicating that the detected levels of expression of these genes reflect biological reality.

Besides defense-related and monoterpene biosynthesis-related gene expression, many genes up-regulated early post-inoculation in CMEN585 concerned cell wall synthesis, general cell growth, and cell proliferation. Since the sampled root tissues included root tips/meristems, it was expected that genes in this category would be expressed. However, these genes were significantly more highly expressed in inoculated roots than in control roots, indicating that an increase in transcription of these genes was a response to *V. dahliae* challenge.

Altogether, the results presented here provide a comprehensive view of gene expression changes in roots and stems of *M. longifolia* in response to *V. dahliae* inoculation and show how different those gene expression profiles are in two species accessions with contrasting wilt susceptibility phenotypes. While adding to the foundational knowledge of mint genetics and disease resistance mechanisms, this work also has a practical objective. We have identified genes that can now be candidates for molecular marker development. Surveys of allelic diversity for *DMR6*-like, *ISPR*-like, and other genes can be conducted in mint germplasm. Molecular markers will ultimately be applied in marker-assisted selection in service of our ultimate goal: breeding new Verticillium wilt-resistant cultivars for the mint industry.

## 4. Materials and Methods

### 4.1. Plant and Fungal Materials

Two *M. longifolia* accessions from South Africa were used in this study. They were obtained from the U.S. Department of Agriculture National Clonal Germplasm Repository in Corvallis, Oregon and maintained in a greenhouse at Oregon State University. CMEN 585 (PI 557767) is considered resistant to Verticillium wilt, as it consistently displays no symptoms or mild symptoms in response to *V. dahliae* inoculation [11]. CMEN 584 (PI 557769) is considered susceptible to Verticillium wilt, as it consistently displays a range of moderate to severe disease symptoms, including stunting, chlorosis, crescent leaf (Figure 1), and ultimately wilting and plant death.

*V. dahliae* isolate was collected from infected mint fields in Oregon as described in [41] and maintained on Czapek-dox agar plates at room temperature until formation of microsclerotia, then at 4 °C.

### 4.2. Fungal Inoculation and RNA Extraction

Two separate experiments were conducted. The first aimed to identify genes differentially expressed in resistant and susceptible mints at the point when the latter began to display disease symptoms, typically by 20 days post-inoculation (dpi). Based on those results, a second experiment was conducted to determine whether any of the same genes were also expressed early in the infection process, up to 24 h post inoculation (hpi).

Stem cuttings of uniform size (3 internodes) from CMEN585 and CMEN584 were planted into autoclaved Sungro^®^ Professional Growing Mix (Sungro Horticulture, Agawam, MA, USA) soil mix and rooted for 2 weeks prior to inoculation in a growth chamber under fluorescent lighting (91 μmol/m^2^) with the following cycle: 22 °C, 10 h light /20 °C, 14 h dark.

In parallel, GFP-labeled *Verticillium dahliae* was grown in Difco™ Czapek-dox broth (Becton, Dickinson and Company, Sparks, MD, USA) on a shaker at 200 RPM at room temperature for 2 weeks. The liquid medium (200 mL) was inoculated with three to four ~0.5 cm^2^ agar plugs from a *V. dahliae* Czapek-dox agar plate. On the day of inoculation, cultures were strained to remove mycelia and centrifuged at 10,000× *g* for 5 min pellet conidia. Conidia were resuspended in H_2_O to the ½ of the original volume of the culture. Counts were done under light microscope using hemocytometer. Conidia were diluted to a final concentration to about 10^7^ conidia/mL.

Root-dip inoculation of rooted cuttings were performed as previously described [11]. Briefly, rooted cuttings were removed from soil and cleaned from debris, and 2–3 plants were submerged into a beaker containing either water or conidial suspension and incubated for 5 min. Inoculated cuttings were randomly replanted in new flats in autoclaved soil and incubated in the growth chamber.

Post inoculation, plants were collected at early and late stages of disease progression.

Root tissue and stem tissue representing the first two internodes above the soil line were collected into separate tubes at four time points post-inoculation: 4 hpi, 24 hpi, 10 dpi, and 20 dpi. Five biological replicate plants were sampled for each genotype for the 4-hpi and 24-hpi time points, and three biological replicate plants were sampled for each genotype at the 10-dpi and 20-dpi time points. Tissue samples were immediately flash-frozen and stored at −80 °C prior to RNA extraction.

Both root and stem plant tissues were ground in liquid N_2_ using mortar and pestle and followed by RNA extraction protocol provided by Qiagen RNeasy Plant Mini Kit (Qiagen, Germantown, MD, USA) with on-column DNA digestion protocol. The RNA concentration was estimated by Nanodrop™ (Thermo Fisher, Waltham, MA, USA), and quality assessment was done using a Bioanalyzer (Agilent, Santa Clara, CA, USA).

### 4.3. Transcriptome Sequencing and Differential Gene Expression Analyses

Sequencing libraries were prepared and Illumina sequencing was performed at the Oregon State University Center for Genome Research and Biocomputing. Samples from the 10-dpi and 20-dpi time points were sequenced on an Illumina HiSeq2000 (Illumina, San Diego, CA, USA) instrument. Samples from the 4-hpi and 24-hpi time points were sequenced on a HiSeq3000 (Illumina, San Diego, CA, USA) instrument.

Transcriptomes of control and inoculated tissues of the two mint genotypes were compared at each time point in pairwise fashion to identify differentially expressed genes. Illumina reads were aligned to the mint reference genome, Mlong585 v3, which was derived from CMEN 585 [42]; (Vining et al., accepted). The HISAT2 pipeline was used for reference-guided alignment, transcript assembly, and derivation of normalized read counts per gene [43]. The R/Bioconducor package DESEQ2 (v. 1.50) was used to detect genes with differential expression. Genes were categorized as differentially expressed if they exhibited a log-fold change absolute value of two or greater and a *p*-value < 0.05. Sets of differentially-expressed genes were analyzed with BLAST2GO version 5 (BioBam) to determine enriched gene ontology categories.

A subset of differentially expressed genes were validated by RT-qPCR. Selection of candidate reference genes from RNA-seq data followed the following criteria: (1) The coefficient variance (cv) values were smaller than 0.3; (2) the calculated Log2FC (fold change) values at each time point were between −0.1 to +0.1; and (3) the base mean values were 500–3000. Beta-glucuronosyltransferase (Mlong585_33938) was ultimately chosen as a reference gene.

Prior to cDNA synthesis a no-RT control was used with all RNA samples to check for DNA contamination. Samples that showed amplification were additionally treated with DNase using DNaseMax kit (Qiagen Germantown, MD, USA). The cDNA was prepared from 1 μg RNA using iScript RT Supermix for RT-qPCR (BioRad, Hercules, CA, USA), following manufacturer’s instructions. Primers’ efficiencies were more than 90%. qPCR was done in a reaction volume of 10 μL containing 1 μL of cDNA, 5 μL of 2 × SYBR SoAdvanced Univ SYBR Green Suprmix (BioRad, Hercules, CA, USA), and 0.5 μL of 10 ng/μL stock concentration of each of forward and reverse primer (Supplemental Table S2 and 3 μL of H_2_O). The reaction was done on Bio-Rad CFX96™ Real-Time PCR System (Bio-Rad, Hercules, CA, USA). The qPCR program consisted of 95 °C for 5 min, then 40 cycles of 94 °C for 15 s, 58 °C for 30 s, and 72 °C for 30 s and a final melt curve step from 65° to 95 °C with a rise of 0.5 °C for 5 s. The reactions were performed in duplicates using three biological replicates. The relative expression level of selected transcripts were normalized with the reference gene and calculated by the comparative ΔΔCt method [44].

## 5. Conclusions

This work provides the first comprehensive survey of gene expression changes in a *Mentha* species in response to *V. dahliae* infection. We identified upregulated genes with known roles in plant defense as well as genes typically associated with monoterpene biosynthesis in glandular trichomes. Paralogs of isopiperitenone reductase genes in particular showed different patterns in root and stem tissues that changed over the course of the experiment. By comparing Verticillium wilt-resistant and wilt-susceptible *M. longifolia* accessions, we uncovered major differences in gene regulation that enabled us to form hypotheses about the mechanisms of Verticillium wilt resistance in mint.

## Figures and Tables

**Table 1:** Numbers of statistically significant differentially expressed genes for two *M. longifolia* genotypes sampled at time points from four hours to 20 days post-inoculation with *Verticillium dahliae*. (*p* < 0.05, logFC > 2).

Genotype	Time Point	Tissue	Up	Down	Total
CMEN 585	4 hpi	root	2138	2339	4477
CMEN 584	4 hpi	root	191	142	333
CMEN 585	24 hpi	root	2278	2410	4688
CMEN 584	24 hpi	root	118	14	132
CMEN 585	10 dpi	root	17	7	24
CMEN 584	10 dpi	root	2	5	7
CMEN 585	20 dpi	root	109	28	137
CMEN 584	20 dpi	root	6	2	8
CMEN 585	4 hpi	stem	26	7	33
CMEN 584	4 hpi	stem	189	441	630
CMEN 585	24 hpi	stem	9	59	68
CMEN 584	24 hpi	stem	18	34	52
CMEN 585	10 dpi	stem	327	234	561
CMEN 584	10 dpi	stem	28	12	40
CMEN 585	20 dpi	stem	152	34	186
CMEN 584	20 dpi	stem	90	37	127

## Data Availability

The *Mentha longifolia* genome sequence used in the analyses presented here can be found at the NCBI Eukaryotic Genomes database under accession number PRJNA310613.

## Supplementary Materials

The following supporting information can be downloaded at: https://www.mdpi.com/article/10.3390/plants11050674/s1, Figure S1: Enriched GO terms; Table S1: Sequence Data Summary; Table S2: RT-qPCR primer sequences.
