# Supplementary material for: Dynamic Tissue—Specific Transcriptome Changes in Response to Verticillium dahliae in Wild Mint Species Mentha longifolia
Source: Plants (Basel). 2022 Mar 1;11(5):674. doi: 10.3390/plants11050674 (PMC8912525; doi:10.3390/plants11050674)
Supplement: Supplementary file 1 [file plants-11-00674-s001.zip › Supplemental_Figure_S1_Enriched_GO_terms.pdf]

Figure S1. Gene Ontology (GO) categories enriched in roots of CMEN 585 at four hours post-inoculation. A. Plant defense; B. Transport; C. Cell Wall Biosynthesis; D. Monoterpene biosynthesis

A.

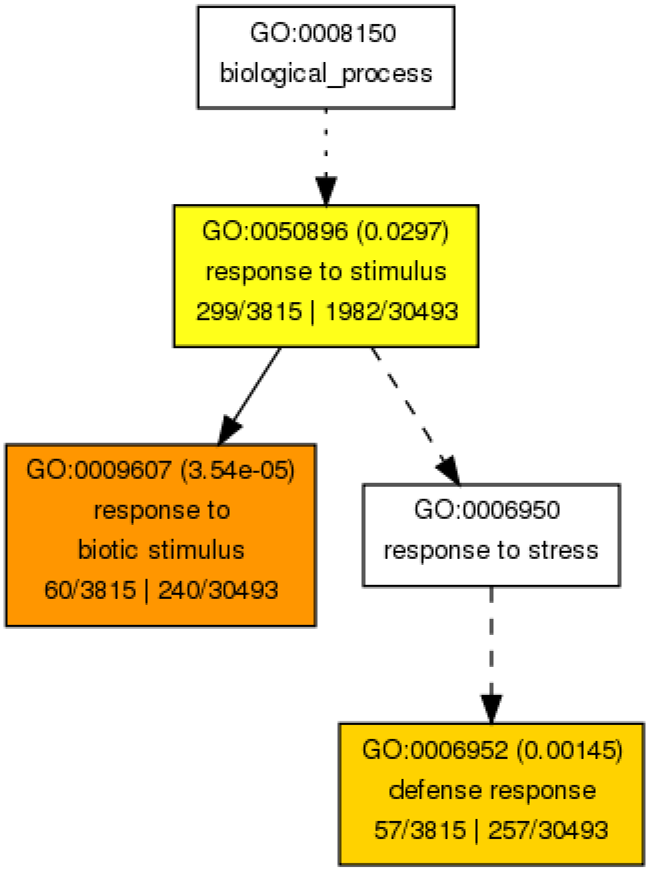

B.

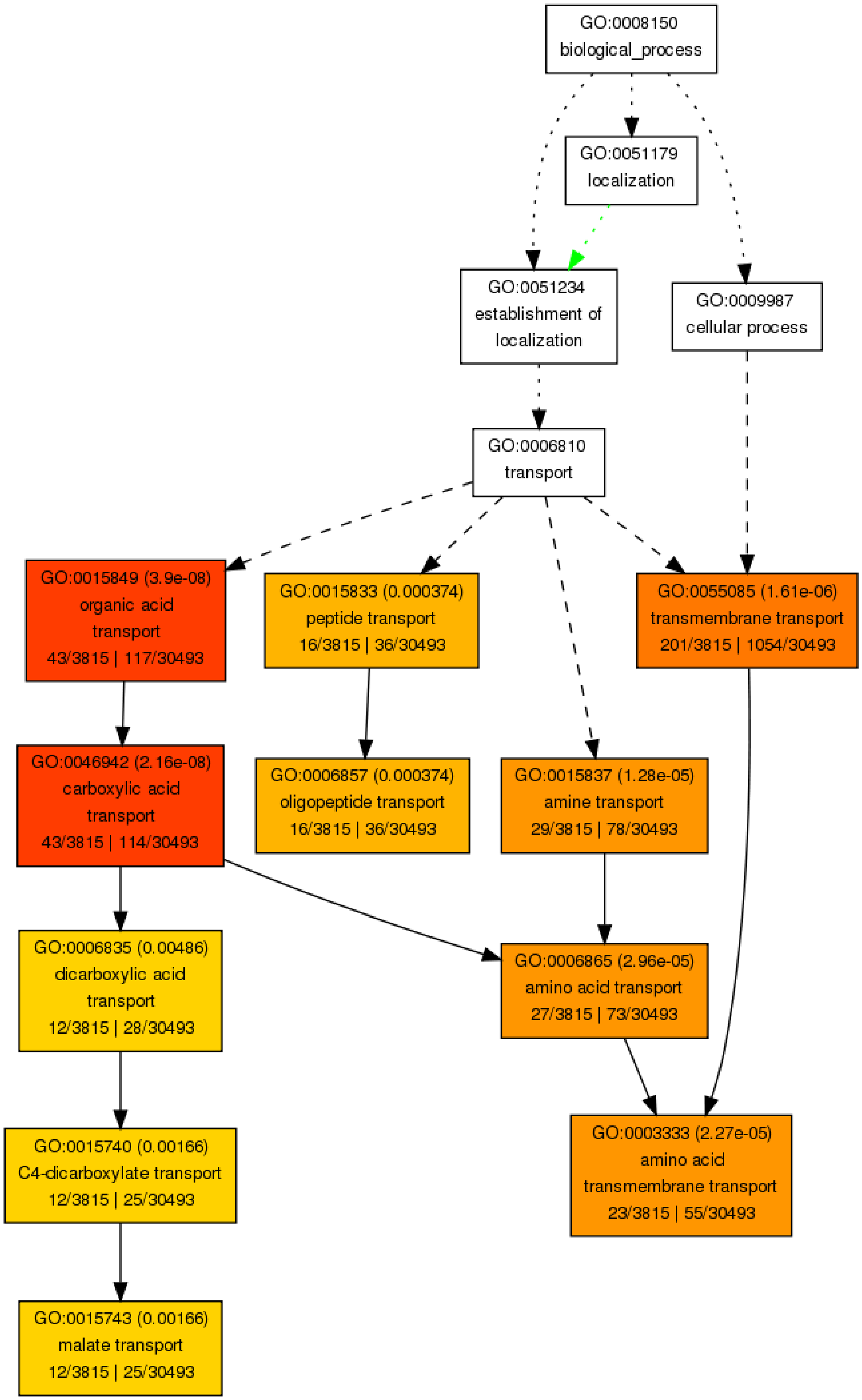

GO:0008150
biological_process
GO:0051179
localization
GO:0051234
establishment of
localization
GO:0009987
cellular process
GO:0006810
transport
GO:0015849 (3.9e-08)
organic acid
transport
43/3815 | 117/30493
GO:0015833 (0.000374)
peptide transport
16/3815 | 36/30493
GO:0055085 (1.61e-06)
transmembrane transport
201/3815 | 1054/30493
GO:0046942 (2.16e-08)
carboxylic acid
transport
43/3815 | 114/30493
GO:0006857 (0.000374)
oligopeptide transport
16/3815 | 36/30493
GO:0015837 (1.28e-05)
amine transport
29/3815 | 78/30493
GO:0006835 (0.00486)
dicarboxylic acid
transport
12/3815 | 28/30493
GO:0006865 (2.96e-05)
amino acid transport
27/3815 | 73/30493
GO:0003333 (2.27e-05)
amino acid
transmembrane transport
23/3815 | 55/30493
GO:0015740 (0.00166)
C4-dicarboxylate transport
12/3815 | 25/30493
GO:0015743 (0.00166)
malate transport
12/3815 | 25/30493

C.

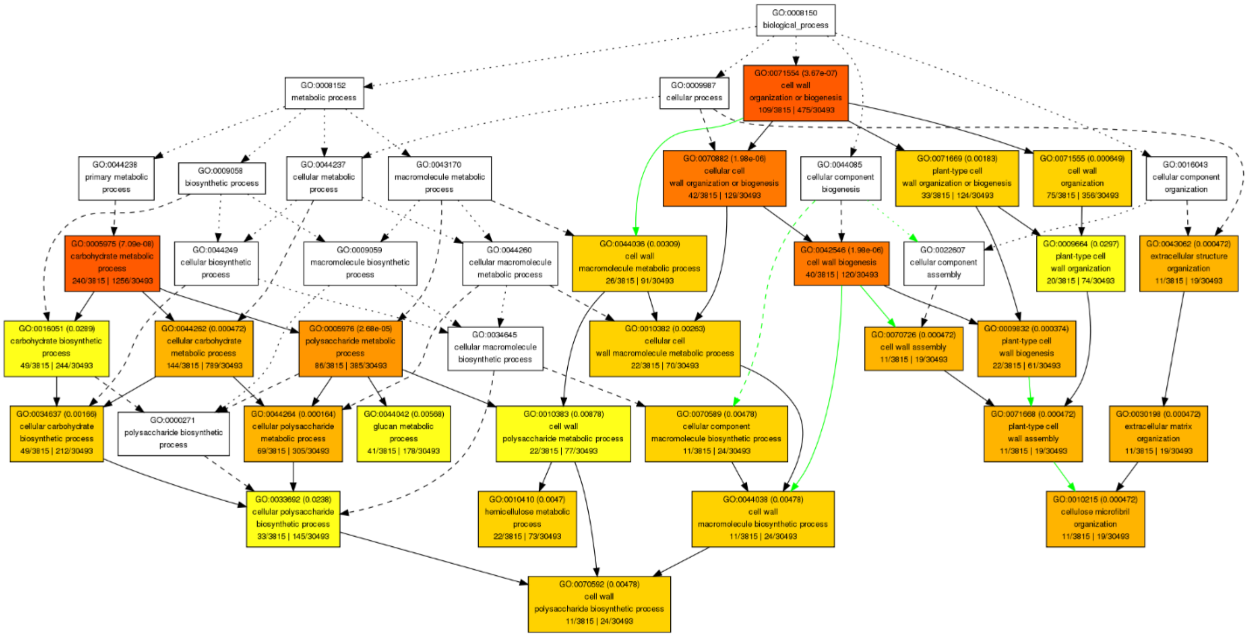
GO:0008150
biological_process
GO:0008152
metabolic process
GO:0009987
cellular process
GO:0071554 (3.67e-07)
cell wall
organization or biogenesis
109/3815 | 475/30493
GO:0044238
primary metabolic
process
GO:0009058
biosynthetic process
GO:0044237
cellular metabolic
process
GO:0043170
macromolecule metabolic
process
GO:0070882 (1.98e-06)
cellular cell
wall organization or biogenesis
42/3815 | 129/30493
GO:0044085
cellular component
biogenesis
GO:0071669 (0.00183)
plant-type cell
wall organization or biogenesis
33/3815 | 124/30493
GO:0071555 (0.000649)
cell wall
organization
75/3815 | 356/30493
GO:0016043
cellular component
organization
GO:0005975 (7.09e-08)
carbohydrate metabolic
process
240/3815 | 1256/30493
GO:0044249
cellular biosynthetic
process
GO:0009059
macromolecule biosynthetic
process
GO:0044260
cellular macromolecule
metabolic process
GO:0044036 (0.00309)
cell wall
macromolecule metabolic process
26/3815 | 91/30493
GO:0042546 (1.98e-06)
cell wall biogenesis
40/3815 | 120/30493
GO:0022607
cellular component
assembly
GO:0009664 (0.0297)
plant-type cell
wall organization
20/3815 | 74/30493
GO:0043062 (0.000472)
extracellular structure
organization
11/3815 | 19/30493
GO:0016051 (0.0289)
carbohydrate biosynthetic
process
49/3815 | 244/30493
GO:0044262 (0.000472)
cellular carbohydrate
metabolic process
144/3815 | 789/30493
GO:0005976 (2.68e-05)
polysaccharide metabolic
process
86/3815 | 385/30493
GO:0034645
cellular macromolecule
biosynthetic process
GO:0010382 (0.00263)
cellular cell
wall macromolecule metabolic process
22/3815 | 70/30493
GO:0070726 (0.000472)
cell wall assembly
11/3815 | 19/30493
GO:0009832 (0.000374)
plant-type cell
wall biogenesis
22/3815 | 61/30493
GO:0034637 (0.00166)
cellular carbohydrate
biosynthetic process
49/3815 | 212/30493
GO:0000271
polysaccharide biosynthetic
process
GO:0044264 (0.000164)
cellular polysaccharide
metabolic process
69/3815 | 305/30493
GO:0044042 (0.00568)
glucan metabolic
process
41/3815 | 178/30493
GO:0010383 (0.00878)
cell wall
polysaccharide metabolic process
22/3815 | 77/30493
GO:0070589 (0.00478)
cellular component
macromolecule biosynthetic process
11/3815 | 24/30493
GO:0071668 (0.000472)
plant-type cell
wall assembly
11/3815 | 19/30493
GO:0030198 (0.000472)
extracellular matrix
organization
11/3815 | 19/30493
GO:0033692 (0.0238)
cellular polysaccharide
biosynthetic process
33/3815 | 145/30493
GO:0010410 (0.0047)
hemicellulose metabolic
process
22/3815 | 73/30493
GO:0044038 (0.00478)
cell wall
macromolecule biosynthetic process
11/3815 | 24/30493
GO:0010215 (0.000472)
cellulose microfibril
organization
11/3815 | 19/30493
GO:0070592 (0.00478)
cell wall
polysaccharide biosynthetic process
11/3815 | 24/30493

D.

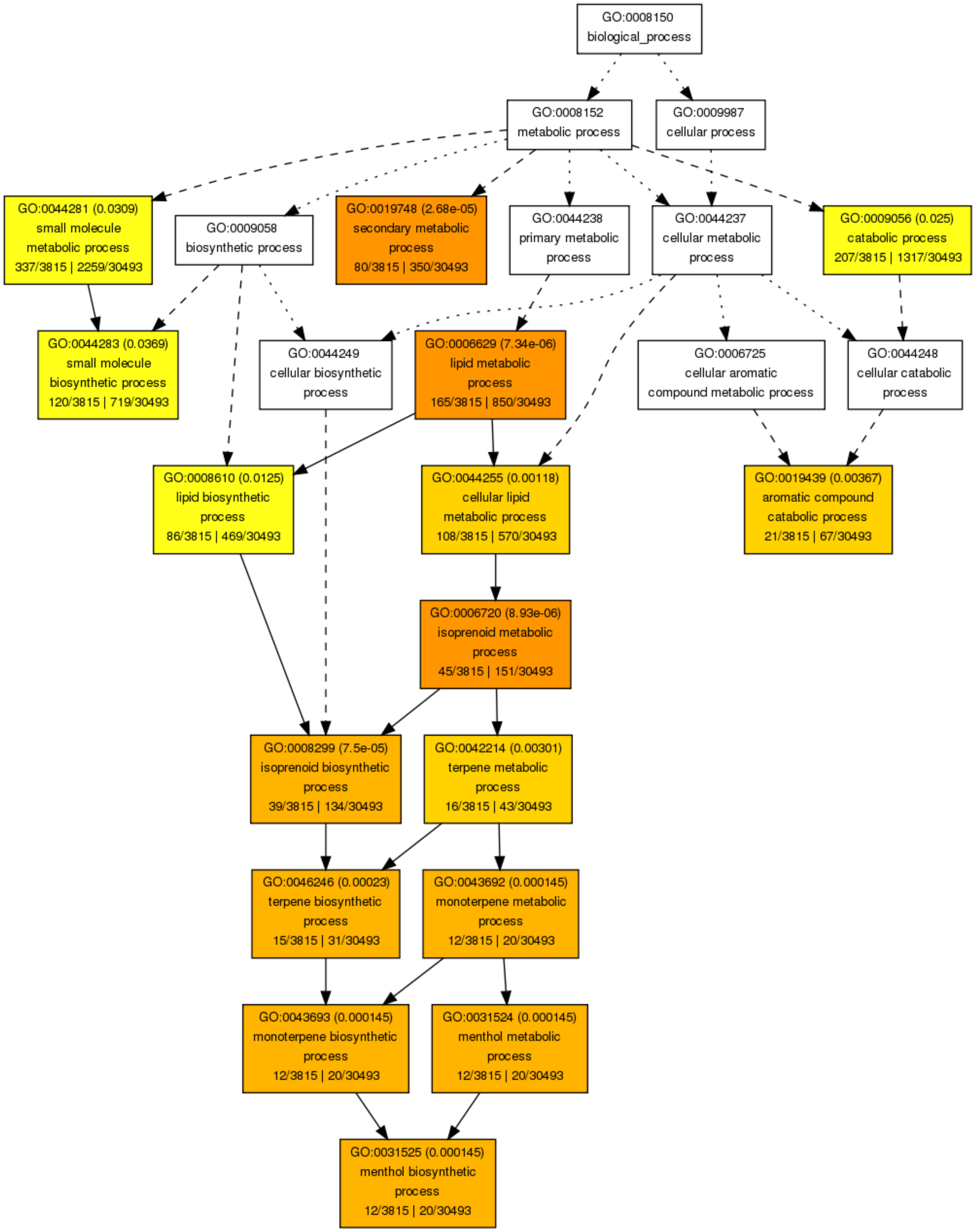
GO:0008150
biological_process
GO:0008152
metabolic process
GO:0009987
cellular process
GO:0044281 (0.0309)
small molecule
metabolic process
337/3815 | 2259/30493
GO:0009058
biosynthetic process
GO:0019748 (2.68e-05)
secondary metabolic
process
80/3815 | 350/30493
GO:0044238
primary metabolic
process
GO:0044237
cellular metabolic
process
GO:0009056 (0.025)
catabolic process
207/3815 | 1317/30493
GO:0044283 (0.0369)
small molecule
biosynthetic process
120/3815 | 719/30493
GO:0044249
cellular biosynthetic
process
GO:0006629 (7.34e-06)
lipid metabolic
process
165/3815 | 850/30493
GO:0006725
cellular aromatic
compound metabolic process
GO:0044248
cellular catabolic
process
GO:0008610 (0.0125)
lipid biosynthetic
process
86/3815 | 469/30493
GO:0044255 (0.00118)
cellular lipid
metabolic process
108/3815 | 570/30493
GO:0019439 (0.00367)
aromatic compound
catabolic process
21/3815 | 67/30493
GO:0006720 (8.93e-06)
isoprenoid metabolic
process
45/3815 | 151/30493
GO:0008299 (7.5e-05)
isoprenoid biosynthetic
process
39/3815 | 134/30493
GO:0042214 (0.00301)
terpene metabolic
process
16/3815 | 43/30493
GO:0046246 (0.00023)
terpene biosynthetic
process
15/3815 | 31/30493
GO:0043692 (0.000145)
monoterpene metabolic
process
12/3815 | 20/30493
GO:0043693 (0.000145)
monoterpene biosynthetic
process
12/3815 | 20/30493
GO:0031524 (0.000145)
menthol metabolic
process
12/3815 | 20/30493
GO:0031525 (0.000145)
menthol biosynthetic
process
12/3815 | 20/30493
